# Presbyopia-correcting performance and subjective outcomes of a trifocal intraocular lens in eyes with different axial lengths: A prospective cohort study

**DOI:** 10.3389/fmed.2022.980110

**Published:** 2022-09-02

**Authors:** Tong Sun, Yiyun Liu, Xiaorui Zhao, Yufei Gao, Tingting Yang, Qianqian Lan, Chuhao Tang, Hong Qi

**Affiliations:** ^1^Department of Ophthalmology, Peking University Third Hospital, Beijing, China; ^2^Beijing Key Laboratory of Restoration of Damaged Ocular Nerve, Peking University Third Hospital, Beijing, China; ^3^Department of Ophthalmology, Affiliated Hospital of Yunnan University, Kunming, China; ^4^Department of Ophthalmology, The People’s Hospital of Guangxi Zhuang Autonomous Region, Nanning, China

**Keywords:** cataract, presbyopia, trifocal intraocular lens, axial length, satisfaction

## Abstract

**Purpose:**

To compare the presbyopia-correcting performance, visual quality, satisfaction and life quality after implantation of a diffractive trifocal intraocular lens (IOL) in eyes with different axial lengths (AL).

**Methods:**

This prospective cohort study enrolled patients with implantation of a trifocal IOL. Manifest refraction, uncorrected and distance-corrected visual acuity at different distances, contrast sensitivity, aberrations and IOL decentration were measured 3 months after surgery. Spectacle independence, adverse photic phenomena, overall satisfaction and life quality were assessed with a questionnaire.

**Results:**

This study included 61 eyes of 61 patients: 16 eyes in the short AL group, 28 eyes in the control group and 17 eyes in the long AL group. Postoperatively, the prediction error (PE) of spherical equivalent showed a difference (*P* = 0.002). The uncorrected near visual acuity in the long AL group was higher (*P* = 0.047). Although a higher IOL decentration was obtained in the long AL group (*P* = 0.034), no significant difference was found in contrast sensitivity and aberrations (all *P* > 0.05). In the questionnaire, patients in the long AL group showed a relatively lower spectacle independence at near distance (*P* = 0.060) and had difficulties in near activities, mental health and role in daily life (*P* = 0.003, 0.021, and 0.033). However, no significant difference was observed in overall satisfaction (*P* = 0.124).

**Conclusion:**

With detailed preoperative evaluation, the trifocal IOL provided satisfactory visual outcomes for patients with different AL. AL had a certain influence on predictability and IOL decentration. And for patients with long AL, the inadaptability to the near focal point might become an important problem.

## Introduction

Presbyopia has become a global problem which was estimated to affect 1.8 billion people worldwide in 2015 (1). It is nearly double the number in 2008 and is still rising (2, 3). With the development of technology, multifocal intraocular lenses (MIOLs) have been successfully correcting presbyopia while treating cataract (4). Through distributing the incoming light to different focal points, MIOLs provide great vision at different distances. However, the visual quality, such as contrast sensitivity, may suffer a certain loss (4, 5).

In a retrospective consecutive case series including about 18,000 cataract operations in 2018, there were approximate 10% of eyes with an axial length (AL) less than 22.5 mm and 10% of eyes with an AL more than 25.5 mm (6). For cataract patients with a short or long AL, the visual outcomes of intraocular lens (IOL) implantation may be limited as a result of the susceptibility to other eye diseases such as strabismus and retinal diseases, lower accuracy of IOL calculation, higher incidences of complications including retinal detachment, IOL decentration and so on (6–9).

The AT LISA tri 839MP (Carl Zeiss Meditec AG) is a diffractive trifocal IOL. With an intermediate focal point at 80 cm and a near focal point at 40 cm, it has achieved a high degree of satisfaction and been widely adopted in the world (10–12). Previous studies have shown that the trifocal IOL can provide satisfactory visual and refractive outcomes in patients with a short or long AL (13–15). But it was still not as good as in the eyes with relatively normal AL (14). Detailed preoperative evaluation and long-term follow-up were also very important (15).

On this basis, this study systematically evaluated the presbyopia-correcting performance, subjective and objective visual quality, postoperative satisfaction and life quality for the trifocal IOL implanted in eyes with different AL.

## Materials and methods

### Patients

This prospective cohort study collected data of patients who underwent bilateral or unilateral cataract surgery with the AT LISA tri 839MP implantation at the Department of Ophthalmology, Peking University Third Hospital, Beijing, China. Surgery was performed between January 2019 and January 2021. This study was consistent with the Declaration of Helsinki for the use of human participants in biomedical research and received the approval of the Ethics Committee of Peking University Third Hospital (IRB00006761-M2019414). An informed consent for surgical procedure and participation in research was obtained from every patient.

For patients with bilateral implantation, only one randomly selected eye was enrolled in this study. Inclusion criteria were patients older than 40 years with significant bilateral or unilateral cataract seeking spectacle independence, with the AL between 21.00 and 30.00 mm, the prediction of postoperative corneal astigmatism less than 1.0 diopters (D), the angle kappa less than 0.50 mm, the corneal spherical aberration less than 0.5 μm and the corneal higher order aberration less than 0.5 μm. Exclusion criteria were patients with serious intraoperative complications, irregular corneal astigmatism, corneal scarring, uveitis, glaucoma, pseudoexfoliation syndrome, macular degeneration or other retinal impairment, amblyopia or patients having difficulties with examinations or 3 months’ follow-up.

Eyes were divided into three groups according to the AL. They were the short AL group (eyes with AL less than 22.50 mm), the control group (eyes with AL between 22.50 and 25.50 mm) and the long AL group (eyes with AL more than 25.50 mm).

### Intraocular lens

The AT LISA tri 839MP is an aspheric trifocal IOL with a combination of diffractive and refractive design. It is single-piece designed, preloaded, and made of hydrophilic acrylic material with a hydrophobic surface. The IOL has a 11.0 mm total diameter, a 4-haptic design and a 6.0 mm biconvex optic, which consists of a central trifocal zone within a diameter of 4.34 mm and a peripheral bifocal zone in the rest. This provides an intermediate addition of + 1.66 D (80 cm) and a near addition of + 3.33 D (40 cm). In theory, the light distribution of far, intermediate and near foci are 50, 20, and 30%. Its available dioptric power ranges from 0.0 to + 32.0 D with increments of 0.5 D (4, 16).

### Preoperative examinations

All patients underwent a thorough preoperative evaluation, including the examination of uncorrected and corrected visual acuity, tonometry, slitlamp examination, manifest refraction, biometric evaluation (IOLMaster 700, Carl Zeiss Meditec AG), corneal topography and aberrations (Pentacam HR, Oculus Optikgerate GmbH), dilated fundoscopy and retinal optical coherence tomography (Cirrus 4000, Carl Zeiss Meditec AG). Visual acuity was recorded in the form of logarithm of the minimum angle of resolution (logMAR). The Holladay 2 formula carried by IOLMaster 700 was applied to calculate the IOL power. All patients received eye drops 3 days before surgery, including 1 drop of levofloxacin and 1 drop of diclofenac sodium 4 times a day.

### Surgical technique

In this study, all surgeries were performed by the same experienced surgeon using topical or retrobulbar anesthesia. For eyes with corneal astigmatism less than 0.50 D, a primary incision at 135° and an auxiliary incision at 45° were used. For the other eyes with higher corneal astigmatism, the primary incision was located at the steep meridian. After the anterior capsulorhexis with a 5.5–6.0 mm diameter, the phacoemulsification and IOL implantation were then performed. The Lumera 700 live microscope (Carl Zeiss Meditec) and the Callisto Eye System (Carl Zeiss Meditec) provided real-time location and eye tracking technique to improve the accuracy of all aspects of surgery. Postoperative medication regimens were consistent in all patients, including 1 drop each of levofloxacin, prednisolone acetate and diclofenac sodium 4 times a day for 1 month. The frequency decreased by 1 time a week.

### Postoperative examinations

Routine examinations including uncorrected visual acuity, tonometry and slitlamp examination were performed at 1 day, 1 week, and 1 month after surgery. Three months after surgery, all patients underwent a comprehensive evaluation, including tonometry, slitlamp examination, manifest refraction, uncorrected and corrected distance (5 m) visual acuity (UDVA, CDVA), uncorrected and distance-corrected intermediate (80 cm) and near (40 cm) visual acuity (UIVA, DCIVA, UNVA, and DCNVA), contrast sensitivity (CS), aberrations and IOL decentration. Besides, patients completed a questionnaire to assess their subjective satisfaction.

**FIGURE 1:**
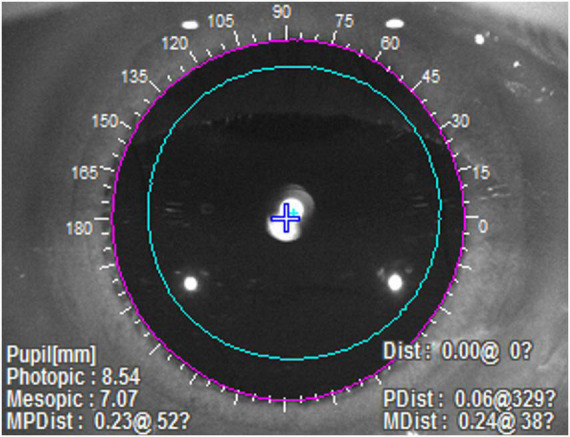
The measurement of IOL decentration with OPD Scan-III. The purple ring represented the automatically recognized edge of the pupil, and the blue ring, which was manually adjusted, represented the edge of the IOL.

Using OPTEC 6500 Vision Tester (Stereo Optical Co., Inc.), the CS was evaluated in four conditions [photopic (85 cd/m^2^), mesopic (3 cd/m^2^), photopic with glare and mesopic with glare] and at five spatial frequencies [1.5, 3, 6, 12, and 18 cycles per degree (cpd)], respectively. The aberrations measured by OPD Scan-III (NIDEK, Inc.) included total aberration, spherical aberration, coma aberration, higher-order aberration, trefoil aberration and tilt aberration. Other indicators reflecting objective visual quality, such as modulation transfer function (MTF) and Strehl ratio (SR), were also evaluated. Besides, OPD Scan-III can automatically recognize and locate the edge of the pupil and measure its position relative to the optic axis (the purple ring in Figure 1). After pupil dilation, the IOL decentration was measured through adjusting the edge of the pupil to the edge of the lens manually (the blue ring in Figure 1). As for the questionnaire, its first part evaluated the spectacle independence at different distances, the incidence of several adverse photic phenomena including glare, halo and starburst, and the overall satisfaction. The second part was the National Eye Institute Visual Function Questionnaire-25 (NEI VFQ-25) in Chinese, a multidimensional questionnaire which applied 25 questions across 12 subscales to assess the general quality of life under the influence of vision problems (17, 18). Patients were asked to assess their life quality after surgery on the eye included in this study.

### Statistical analysis

Continuous variables were recorded as means and standard deviations (mean ± SD), and categorical variables as counts and percentages. Statistical analysis was performed with SPSS Statistics for Windows software (version 22.0, IBM Corp.). For preoperative and postoperative examination results, Kolmogorov-Smirnov test was used to verify the normal distribution. Then one-way ANOVA or Kruskal-Wallis H test was used to compare the means. For the percentages corresponding to each answer to items in the questionnaire, Chi-square test or Fisher exact test was used. A *P*-value less than 0.05 was considered statistically significant.

## Results

This study included 61 eyes of 61 patients (27 males and 34 females), and the mean age was 65.6 ± 13.5 years. 16 patients were in the short AL group, 28 patients in the control group, and 17 patients in the long AL group. All patients were followed up for 3 months after surgery. Table 1 shows the preoperative characteristics. There were statistically significant differences in AL, preoperative spherical equivalent (SE), IOL power and target SE (all *P* < 0.001). No significant difference was found in sex ratio, age, UDVA, CDVA, corneal astigmatism, and angle kappa (*P* = 0.113, 0.053, 0.303, 0.807, 0.148, and 0.294).

**TABLE 1:** Demographics and preoperative characteristics.

Parameter	Short AL group	Control group	Long AL group	*P-value*
Sex (male/female)	4/12	16/12	7/10	0.113
Age (years)	66.2 ± 10.0	69.4 ± 13.3	59.9 ± 12.8	0.053
UDVA (logMAR)	0.33 ± 0.32	0.47 ± 0.29	0.50 ± 0.45	0.303
CDVA (logMAR)	0.22 ± 0.20	0.28 ± 0.26	0.29 ± 0.27	0.807
SE (D)	0.13 ± 2.01	−1.34 ± 3.27^c^	−9.55 ± 5.97^b^	< 0.001*
AL (mm)	21.80 ± 0.38^b,c^	23.73 ± 0.76^a,c^	27.15 ± 1.29^a,b^	< 0.001*
Corneal astigmatism (D)	0.56 ± 0.21	0.74 ± 0.36	0.61 ± 0.27	0.148
Angle kappa (mm)	0.22 ± 0.11	0.30 ± 0.18	0.26 ± 0.16	0.294
IOL power (D)	23.6 ± 1.91^b,c^	20.0 ± 2.19^a,c^	11.2 ± 3.79^a,b^	< 0.001*
Target SE (D)	0.06 ± 0.14^b,c^	−0.10 ± 0.18^a,c^	−0.27 ± 0.19^a,b^	< 0.001*

One-way ANOVA test or Kruskal-Wallis H test; Chi-square test for the sex ratio.

ACD, anterior chamber depth; AL, axial length; CDVA, corrected distance visual acuity; D, diopter; IOL, intraocular lens; logMAR, logarithm of the minimum angle of resolution; SE, spherical equivalent; UDVA, uncorrected distance visual acuity.

^a^P < 0.05 vs. the short AL group; ^b^P < 0.05 vs. the control group; ^c^P < 0.05 vs. the long AL group.

*P < 0.05 among three groups.

### Efficacy

**FIGURE 2:**
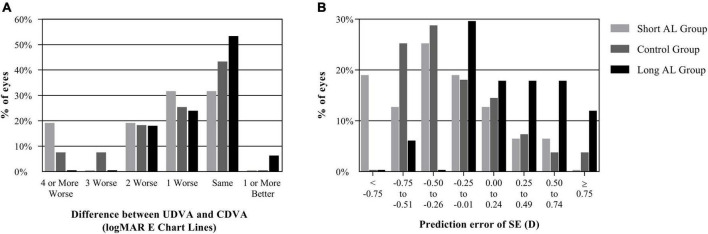
The refractive outcomes of three groups. **(A)** Difference between UDVA and CDVA; **(B)** prediction error of SE. (AL, axial length; CDVA, corrected distance visual acuity; logMAR, logarithm of the minimum angle of resolution; SE, spherical equivalent; UDVA, un corrected distance visual acuity).

To show the refractive efficacy, Figure 2A showed the difference between postoperative UDVA and CDVA. There were respectively 31, 43, and 59% of eyes with UDVA same or better than its CDVA (*P* = 0.272). The proportions of eyes whose UDVA was within 1 line of CDVA were 63, 68, and 82% in the three groups (*P* = 0.425).

### Safety

The preoperative CDVA in the three groups were 0.22 ± 0.20 logMAR, 0.28 ± 0.26 logMAR and 0.29 ± 0.27 logMAR. The postoperative CDVA were 0.05 ± 0.08 logMAR, 0.02 ± 0.04 logMAR and 0.02 ± 0.04 logMAR. The postoperative CDVA was significantly higher than that preoperatively in every group (all *P* < 0.05). Specially, there was no report about eyes with worse CDVA 3 months after surgery. In a long-term follow-up for a year, only one patient in the long AL group (5.9%) suffered from serious IOL decentration.

### Predictability

As is shown in Table 2, the mean postoperative SE were similar among the three groups (*P* = 0.227). There was also no significant difference in the postoperative cylinder (*P* = 0.070). Compared to the target SE predicted preoperatively, the prediction error (PE) in the long AL group was significantly different from that in the other two groups (*P* = 0.002). Figure 2B showed the distribution of PE. There were 31, 32, and 47% of eyes with a PE from -0.25 to 0.24 D in the three groups (*P* = 0.538). However, the proportions of eyes with a PE from -0.75 to -0.26D were 38, 54, and 6% (*P* = 0.005), and the proportions of eyes with a PE from 0.25 to 0.74 D were 13, 11, and 35% (*P* = 0.129). There was no significant difference in the absolute value of PE among the three groups (*P* = 0.349).

**TABLE 2:** Refraction and visual acuity.

Parameter	Short AL group	Control group	Long AL group	*P-value*
Postoperative SE (D)	−0.29 ± 0.57	−0.29 ± 0.43	−0.06 ± 0.38	0.227
Postoperative cylinder (D)	0.67 ± 0.44	0.70 ± 0.39	0.44 ± 0.30	0.070
PE (D)	−0.35 ± 0.52^c^	−0.19 ± 0.41^c^	0.21 ± 0.43^a,b^	0.002*
Absolute value of PE (D)	0.50 ± 0.38	0.37 ± 0.25	0.38 ± 0.27	0.349
UDVA (logMAR)	0.13 ± 0.13	0.08 ± 0.09	0.05 ± 0.07	0.108
CDVA (logMAR)	0.05 ± 0.08	0.02 ± 0.04	0.02 ± 0.04	0.837
UIVA (logMAR)	0.09 ± 0.10	0.09 ± 0.10	0.07 ± 0.08	0.842
DCIVA (logMAR)	0.10 ± 0.09	0.13 ± 0.08	0.11 ± 0.11	0.474
UNVA (logMAR)	0.18 ± 0.10^c^	0.13 ± 0.08	0.08 ± 0.11^a^	0.047*
DCNVA (logMAR)	0.12 ± 0.08	0.11 ± 0.08	0.07 ± 0.08	0.160

One-way ANOVA test or Kruskal-Wallis H test.

AL, axial length; CDVA, corrected distance visual acuity; D, diopter; DCIVA, distance-corrected intermediate visual acuity; DCNVA, distance-corrected near visual acuity; logMAR, logarithm of the minimum angle of resolution; PE, prediction error; SE, spherical equivalent; UDVA, uncorrected distance visual acuity; UIVA, uncorrected intermediate visual acuity; UNVA, uncorrected near visual acuity.

^a^P < 0.05 vs. the short AL group; ^b^P < 0.05 vs. the control group; ^c^P < 0.05 vs. the long AL group.

*P < 0.05 among three groups.

### Visual acuity at different distances

Table 2 shows the uncorrected and distance-corrected visual acuity at different distances. No significant difference was found in the UDVA and UIVA among the three groups (*P* = 0.108 and 0.842). While the UNVA in the long AL group was significantly higher than that in the short AL group (*P* = 0.047). No marked difference was found in the CDVA, DCIVA, and DCNVA among three groups (*P* = 0.837, 0.474, and 0.160).

### Contrast sensitivity and aberrations

**FIGURE 3:**
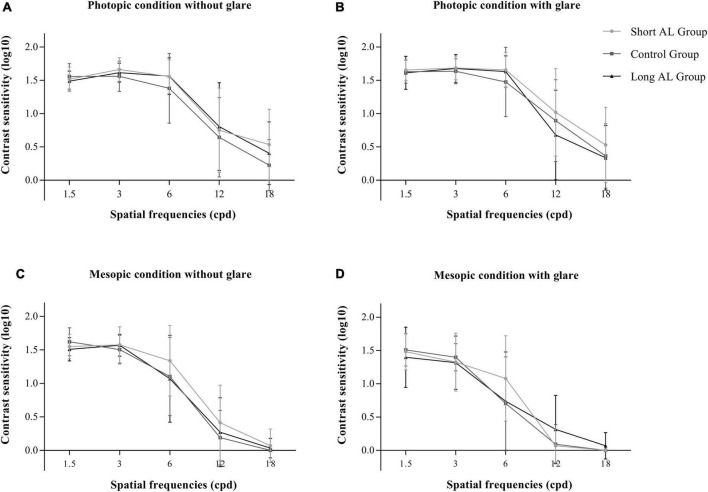
The contrast sensitivity of three groups. **(A)** Photopic condition without glare; **(B)** photopic condition with glare; **(C)** mesopic condition without glare; **(D)** mesopic condition with glare. (AL, axial length; cpd, cycle per degree).

The CS was recorded as the log10 value. Figure 3 shows the CS under different conditions and at different spatial frequencies. No significant difference was observed among the three groups (all *P* > 0.05). In addition, Aberrations, MTF and SR for a 4 mm pupil were measured using OPD Scan-III (Table 3). The MTF was recorded as the ratio to normal. There was no significant difference in these indicators among the three groups (all *P* > 0.05).

**TABLE 3:** Aberrations, MTF, SR and IOL decentration using OPD scan-III.

Parameter	Short AL group	Control group	Long AL group	*P-value*
Total aberration (μm)	0.56 ± 0.20	0.63 ± 0.22	0.58 ± 0.21	0.586
Tilt aberration (μm)	0.19 ± 0.14	0.23 ± 0.14	0.26 ± 0.20	0.616
Higher-order aberration (μm)	0.29 ± 0.07	0.28 ± 0.12	0.29 ± 0.16	0.956
Coma aberration (μm)	0.09 ± 0.07	0.09 ± 0.07	0.08 ± 0.09	0.876
Trefoil aberration (μm)	0.25 ± 0.07	0.24 ± 0.13	0.23 ± 0.15	0.874
Spherical aberration (μm)	0.03 ± 0.02	0.02 ± 0.02	0.03 ± 0.03	0.969
MTF	36.32 ± 7.42	35.70 ± 10.17	39.22 ± 12.16	0.570
SR	0.05 ± 0.02	0.04 ± 0.03	0.05 ± 0.04	0.637
IOL decentration (mm)	0.17 ± 0.07^c^	0.25 ± 0.13	0.28 ± 0.16^a^	0.085

One-way ANOVA test or Kruskal-Wallis H test.

AL, axial length; D, diopter; IOL, intraocular lens; MTF, modulation transfer function; SR, Strehl ratio.

^a^P < 0.05 vs. the short AL group; ^b^P < 0.05 vs. the control group; ^c^P < 0.05 vs. the long AL group.

*P < 0.05 among three groups.

### Intraocular lens decentration

As is shown in Table 3, the IOL decentration were 0.17 ± 0.07, 0.25 ± 0.13, and 0.28 ± 0.16 mm. Among the three groups, there was a tendency that the longer the AL, the higher the IOL decentration was (*P* = 0.085). More than that, a significantly higher IOL decentration was obtained in the long AL group than in the short AL group in the multiple comparisons (*P* = 0.034).

### Satisfaction

The first part of the questionnaire was the evaluation of spectacle independence, adverse photic phenomena and overall satisfaction for the eye included in this study (Table 4). The proportion of patients who achieved spectacle independence at far, intermediate and near distances were similar among the three groups (*P* = 0.999, 0.803, and 0.060). There was also no significant difference in the incidence of glare, halo and starburst among them (*P* = 0.927, 0.558, and 0.141). As shown in Table 4, patients were given five options to assess their satisfaction with the eye included and corresponding scores were calculated. Although the proportions of patients choosing “neutral” were different among the three groups (*P* = 0.033), the other choices (*P* = 0.671, 0.678, and 0.740) and the average score (*P* = 0.488) were still similar.

**TABLE 4:** Spectacle independence, adverse photic phenomena and overall satisfaction.

Parameter	Short AL group	Control group	Long AL group	*P-value*
Spectacle independence				
Far	15 (93.75%)	25 (89.29%)	16 (94.12%)	0.999
Intermediate	15 (93.75%)	27 (96.43%)	15 (88.24%)	0.803
Near	16 (100.00%)	22 (78.57%)	12 (70.59%)	0.060
Adverse photic phenomena				
Glare	4 (25.00%)	6 (21.43%)	5 (29.41%)	0.927
Halo	4 (25.00%)	8 (28.57%)	7 (41.18%)	0.558
Starburst	7 (43.75%)	6 (21.43%)	8 (47.06%)	0.141
Satisfaction score				
5 (very satisfied)	8 (50.00%)	11 (39.29%)	6 (35.29%)	0.671
4 (satisfied)	7 (43.75%)	15 (53.57%)	7 (41.18%)	0.678
3 (neutral)	0 (0.00%)	1 (3.57%)	4 (23.53%)	0.033*
2 (dissatisfied)	1 (6.25%)	1 (3.57%)	0 (0.00%)	0.740
1 (very dissatisfied)	0 (0.00%)	0 (0.00%)	0 (0.00%)	/
Average score	4.38 ± 0.81	4.29 ± 0.71	4.12 ± 0.78	0.488

Chi-square test or Fisher exact test; Kruskal-Wallis H test for the average satisfaction score.

AL, axial length.

*P < 0.05 among three groups.

### Life quality

The second part of subjective outcomes was the NEI VFQ-25 questionnaire. The average score of its 12 scales and the total score were shown in Table 5. Patients in the long AL group might have difficulties in near activities, mental health and role in daily life (*P* = 0.003, 0.021, and 0.033). Patients in the short AL group might also have a poor performance in social functioning and role in daily life (*P* = 0.049 and 0.033). However, no significant difference was observed in the total score of the general life quality among the three groups (*P* = 0.124).

**TABLE 5:** General life quality with NEI VFQ-25.

Scale	Short AL group	Control group	Long AL group	*P-value*
General health	70.31 ± 31.91	53.85 ± 23.12	51.79 ± 20.72	0.094
General vision	90.00 ± 10.38	92.31 ± 9.92	85.71 ± 12.23	0.232
Ocular pain	72.66 ± 19.48	82.69 ± 18.73	81.25 ± 12.74	0.149
Near activities	86.11 ± 23.29^c^	92.63 ± 20.01^c^	75.60 ± 21.30^a,b^	0.003*
Distance activities	90.28 ± 17.23	92.63 ± 12.54	91.96 ± 9.87	0.712
Social functioning	93.33 ± 10.42^b^	99.04 ± 3.40^a^	98.21 ± 6.68	0.049*
Mental health	85.16 ± 12.26	89.42 ± 13.44^c^	77.23 ± 22.81^b^	0.021*
Role difficulties	68.75 ± 30.62^b^	87.50 ± 20.62^a,c^	73.21 ± 22.92^b^	0.033*
Dependency	90.63 ± 12.50	94.87 ± 12.26	86.90 ± 27.29	0.361
Driving	89.29 ± 7.93	85.42 ± 23.07	77.78 ± 31.46	0.721
Color vision	93.33 ± 17.59	100.00 ± 0.00	94.64 ± 14.47	0.161
Peripheral vision	88.33 ± 20.85	99.04 ± 4.90	94.64 ± 14.47	0.094
Total score	83.90 ± 14.51	89.15 ± 10.19	82.53 ± 10.55	0.124

Kruskal-Wallis H test.

AL, axial length; D, diopter; IOL, intraocular lens; MTF, modulation transfer function; SR, Strehl ratio.

^a^P < 0.05 vs. the short AL group; ^b^P < 0.05 vs. the control group; ^c^P < 0.05 vs. the long AL group.

*P < 0.05 among three groups.

## Discussion

Today, cataract patients have been increasingly demanding better visual quality and spectacle independence after surgery. The AT LISA tri 839MP IOL minimizes the loss of visual quality while providing reliable far, intermediate and near visual acuity. It was proved to provide good effectiveness, predictability, stability, and safety for patients in previous studies (11, 19, 20). There is an unignorable proportion of patients with relatively short or long AL, who had strong demand, but hesitated regarding trifocal IOL implantation as a result of potential eye diseases and calculation errors. In addition, since these patients wear glasses for years, implantation of the trifocal IOL to obtain spectacle independence may provide them greater benefits.

The grouping criteria of AL in this study were based on a large sample study in 2018 (6). There was no significant difference in demographics and preoperative characteristics except AL, SE, IOL power and target SE among the three groups, which indicated that no significant preoperative bias was observed. Three months after surgery, the trifocal IOL in all the three groups achieved good efficacy and safety. As the logMAR E chart had a more detailed grade of vision, such as the line of 0.05 logMAR and 0.15 logMAR, the proportions of eyes whose UDVA was within 1 line of CDVA in this study were then relatively low in this study. As for the predictability, the Holladay 2 formula had a tendency of myopia drift in eyes with short AL and a contrary tendency in eyes with long AL in previous studies (6, 21). Therefore, the target SE was adjusted according to the AL of each eye. Three months after surgery, the postoperative SE in the three groups were similar, and the PE showed positive correlation with AL. This was also confirmed by the difference in PE distribution in Figure 2A. Savini et al. summed up previous studies and concluded that the refractive accuracy of IOL power calculation in short AL eyes (AL < 22 mm) is still relatively lower (22). But no significant difference was observed in the absolute of PE, representing the degree of the refraction drift.

In the uncorrected and corrected visual acuity at different distances, UNVA was the only one that showed significant difference. Combined with the fact that there was no significant difference in DCNVA, refractive drift was considered to be the main cause. As is shown in Table 2, it seemed contradictory that the long AL group achieved a better UNVA with a lower degree of myopia. However, the near visual acuity was measured at 40 cm in this study, which was consistent with the near focus of the trifocal IOL (4). For this reason, with the postoperative SE closer to emmetropia, the trifocal IOL in the long AL group performed better at 40 cm. To sum up, the trifocal IOL provided satisfactory visual and refractive outcomes for patients with short or long AL, but the adjustment of the target SE was of vital importance.

Contrast sensitivity reflected subjective visual quality, while aberrations, MTF and SR reflected objective visual quality. In this study, the difference in AL did not significantly affect the subjective or objective visual quality postoperatively. IOL decentration was an important factor influencing postoperative visual quality (23). Montes-Mico et al. showed that the visual quality of a diffractive bifocal IOL remained stable with a decentration less than 0.4 mm (24). While Xu et al. indicated that the MTF, PSF, and coma aberration of another diffractive bifocal IOL were affected when the IOL decentration higher than 0.25 mm (25). The IOL decentration of the three groups were all within an acceptable range and aberrations, MTF and SR showed no significant difference. But the tendency of IOL decentration increasing with the increase of AL should be paid more attention. Previous studies have demonstrated it in IOLs with a C-loop haptic design (8, 26). Few studies have systematically analyzed the patterns in IOLs with a plate-haptic design (27).

To assess subjective outcomes of the trifocal IOL, spectacle independence, adverse photic phenomena including glare, halo and starburst, overall satisfaction, and general life quality were recorded. All three groups obtained reliable outcomes in the occurrence of adverse photic phenomena, which was consistent with previous studies (13, 14). As mentioned above, patients in the long AL group had a better UNVA at 40 cm, but they obtained relatively lower spectacle independence when looking at something close by. This might because that these patients with high myopia were used to a shorter reading distance (28, 29). Beyond that, in the analysis of general life quality in different aspects, patients in the long AL group had a statistically worse experience in near activities, mental health and role functioning. This might suggest that patients with long AL were uncomfortable with the near focus point at 40 cm provided by the trifocal IOL for close reading and work (30). Sezgin Asena et al. had concluded that different intermediate focal points could meet different needs postoperatively (10). But there were few studies about the visual outcomes and satisfaction of MIOLs with different near focal points. In addition, although the visual acuity, spectacle independence and satisfaction showed no obvious deficiencies, a similar situation had also occurred in patients in the short AL group, with a statistically lower evaluation in role difficulties and social functioning. Patients with short or long AL might undergo a longer adjustment process to new reading habits provided by the trifocal IOL after surgery. However, the difference in this area was not large enough to cause a change in overall satisfaction.

The most important limitation of the current study is the limited follow-up time. To further clarify the adaptation process after the trifocal IOL implantation, a longer follow-up and the comparison of outcomes at each stage were necessary. A second limitation of the study is the relatively small sample size, which was as a result of the strict inclusion criteria. Only one eye of each patient was included to reduce potential bias.

## Conclusion

This study systematically evaluated the presbyopia-correcting performance, visual quality and subjective satisfaction of eyes with different AL after the implantation of AT LISA tri 839MP. With detailed preoperative evaluation and postoperative follow-up, the trifocal IOL provided stable and satisfactory visual outcomes for patients with short or long AL. However, the AL had a certain influence on the predictability of IOL calculation and IOL decentration. Besides, the inadaptability to the near focal point of patients with long AL might become an unignorable problem for the application of the trifocal IOL in these patients. This is of great significance for subsequent studies on neural adaptation of multifocal IOLs.

## Data availability statement

The raw data supporting the conclusions of this article will be made available by the authors, without undue reservation.

## Ethics statement

The studies involving human participants were reviewed and approved by the Ethics Committee of Peking University Third Hospital. The patients/participants provided their written informed consent to participate in this study.

## Author contributions

TS, YL, and HQ were responsible for the study design and initial plan. TS, YG, and TY involved in patient examination and data collection. TS and XZ were responsible for data interpretation, statistical analysis, and manuscript drafting. QL and CT improved the study protocol and supervised the study. HQ was the guarantor for this article. All authors contributed to the article and approved the submitted version.

## References

[1] Fricke TR Tahhan N Resnikoff S Papas E Burnett A Ho SM Global prevalence of presbyopia and vision impairment from uncorrected presbyopia: systematic review, meta-analysis, and modelling. *Ophthalmology.* (2018) 125:1492–9. 10.1016/j.ophtha.2018.04.013 29753495

[2] Holden BA Fricke TR Ho SM Wong R Schlenther G Cronjé S Global vision impairment due to uncorrected presbyopia. *Arch Ophthalmol.* (2008) 126:1731–9. 10.1001/archopht.126.12.1731 19064856

[3] Wolffsohn JS Davies LN. Presbyopia: effectiveness of correction strategies. *Prog Retin Eye Res.* (2019) 68:124–43. 10.1016/j.preteyeres.2018.09.004 30244049

[4] Rampat R Gatinel D. Multifocal and extended depth-of-focus intraocular lenses in 2020. *Ophthalmology.* (2020) 128:e164–85. 10.1016/j.ophtha.2020.09.026 32980397

[5] Alio JL Plaza-Puche AB Fernandez-Buenaga R Pikkel J Maldonado M. Multifocal intraocular lenses: an overview. *Surv Ophthalmol.* (2017) 62:611–34. 10.1016/j.survophthal.2017.03.005 28366683

[6] Melles RB Holladay JT Chang WJ. Accuracy of intraocular lens calculation formulas. *Ophthalmology.* (2018) 125:169–78. 10.1016/j.ophtha.2017.08.027 28951074

[7] van Leeuwen R Haarman AEG van de Put MAJ Klaver CCW Los LI. Dutch rhegmatogenous retinal detachment study g. association of rhegmatogenous retinal detachment incidence with myopia prevalence in the Netherlands. *JAMA Ophthalmol.* (2021) 139:85–92. 10.1001/jamaophthalmol.2020.5114 33237293 PMC7689575

[8] Zhu X He W Zhang Y Chen M Du Y Lu Y. Inferior decentration of multifocal intraocular lenses in myopic eyes. *Am J Ophthalmol.* (2018) 188:1–8. 10.1016/j.ajo.2018.01.007 29355482

[9] Qureshi MH Steel DHW. Retinal detachment following cataract phacoemulsification-a review of the literature. *Eye.* (2020) 34:616–31. 10.1038/s41433-019-0575-z 31576027 PMC7093479

[10] Sezgin Asena B. Visual and refractive outcomes, spectacle independence, and visual disturbances after cataract or refractive lens exchange surgery: comparison of 2 trifocal intraocular lenses. *J Cataract Refract Surg.* (2019) 45:1539–46. 10.1016/j.jcrs.2019.06.005 31587938

[11] Ganesh S Brar S Pawar A. Long-term visual outcomes and patient satisfaction following bilateral implantation of trifocal intraocular lenses. *Clin Ophthalmol.* (2017) 11:1453–9. 10.2147/OPTH.S125921 28860693 PMC5558567

[12] Ruiz-Alcocer J Madrid-Costa D Garcia-Lazaro S Ferrer-Blasco T Montes-Mico R. Optical performance of two new trifocal intraocular lenses: through-focus modulation transfer function and influence of pupil size. *Clin Exp Ophthalmol.* (2014) 42:271–6. 10.1111/ceo.12181 23927051

[13] Alfonso JF Fernandez-Vega-Cueto A Alfonso-Bartolozzi B Rodriguez-Una I Montes-Mico R. Visual and refractive outcomes in hyperopic pseudophakic patients implanted with a trifocal intraocular lens. *Clin Ophthalmol.* (2019) 13:2261–8. 10.2147/OPTH.S229228 31819350 PMC6875502

[14] Steinwender G Schwarz L Bohm M Slavik-Lencova A Hemkeppler E Shajari M Visual results after implantation of a trifocal intraocular lens in high myopes. *J Cataract Refract Surg.* (2018) 44:680–5. 10.1016/j.jcrs.2018.04.037 29909961

[15] Javaloy J Rivera E Montalban R Beltran J Munoz G Rohrweck S. Diffractive trifocal pseudophakic intraocular lenses in high myopic eyes: 2-year assessment after implantation. *Graefes Arch Clin Exp Ophthalmol.* (2019) 257:1331–9. 10.1007/s00417-019-04302-5 30968291

[16] Webers VSC Bauer NJC Saelens IEY Creten OJM Berendschot T van den Biggelaar F Comparison of the intermediate distance of a trifocal IOL with an extended depth-of-focus IOL: results of a prospective randomized trial. *J Cataract Refract Surg.* (2020) 46:193–203. 10.1097/j.jcrs.0000000000000012 32126031

[17] Wan Y Zhao L Huang C Xu Y Sun M Yang Y Validation and comparison of the national eye institute visual functioning questionnaire-25 (NEI VFQ-25) and the Visual Function Index-14 (VF-14) in patients with cataracts: a multicentre study. *Acta Ophthalmol.* (2021) 99:e480–8. 10.1111/aos.14606 32940410 PMC8359188

[18] Marella M Pesudovs K Keeffe JE O’Connor PM Rees G Lamoureux EL. The psychometric validity of the NEI VFQ-25 for use in a low-vision population. *Invest Ophthalmol Vis Sci.* (2010) 51:2878–84. 10.1167/iovs.09-4494 20089878

[19] Bohm M Petermann K Hemkeppler E Kohnen T. Defocus curves of 4 presbyopia-correcting IOL designs: diffractive panfocal, diffractive trifocal, segmental refractive, and extended-depth-of-focus. *J Cataract Refract Surg.* (2019) 45:1625–36. 10.1016/j.jcrs.2019.07.014 31706517

[20] Mencucci R Favuzza E Caporossi O Savastano A Rizzo S. Comparative analysis of visual outcomes, reading skills, contrast sensitivity, and patient satisfaction with two models of trifocal diffractive intraocular lenses and an extended range of vision intraocular lens. *Graefes Arch Clin Exp Ophthalmol.* (2018) 256:1913–22. 10.1007/s00417-018-4052-3 29980919

[21] Cooke DL Cooke TL. A comparison of two methods to calculate axial length. *J Cataract Refract Surg.* (2019) 45:284–92. 10.1016/j.jcrs.2018.10.039 30851805

[22] Savini G Taroni L Hoffer KJ. Recent developments in intraocular lens power calculation methods-update 2020. *Ann Transl Med.* (2020) 8:1553. 10.21037/atm-20-2290 33313298 PMC7729321

[23] Ashena Z Maqsood S Ahmed SN Nanavaty MA. Effect of intraocular lens tilt and decentration on visual acuity, dysphotopsia and wavefront aberrations. *Vision.* (2020) 4:41. 10.3390/vision4030041 32937750 PMC7559075

[24] Montes-Mico R Lopez-Gil N Perez-Vives C Bonaque S Ferrer-Blasco T. In vitro optical performance of nonrotational symmetric and refractive-diffractive aspheric multifocal intraocular lenses: impact of tilt and decentration. *J Cataract Refract Surg.* (2012) 38:1657–63. 10.1016/j.jcrs.2012.03.040 22819523

[25] Xu J Zheng T Lu Y. Effect of decentration on the optical quality of monofocal, extended depth of focus, and bifocal intraocular lenses. *J Refract Surg.* (2019) 35:484–92. 10.3928/1081597X-20190708-02 31393986

[26] Gu X Chen X Yang G Wang W Xiao W Jin G Determinants of intraocular lens tilt and decentration after cataract surgery. *Ann Transl Med.* (2020) 8:921. 10.21037/atm-20-1008 32953721 PMC7475414

[27] Zhu X Meng J He W Rong X Lu Y. Comparison of the rotational stability between plate-haptic toric and C-loop haptic toric IOLs in myopic eyes. *J Cataract Refract Surg.* (2020) 46:1353–9. 10.1097/j.jcrs.0000000000000259 33060472

[28] Attia MS Auffarth GU Khoramnia R Linz K Kretz FT. Near and intermediate reading performance of a diffractive trifocal intraocular lens using a reading desk. *J Cataract Refract Surg.* (2015) 41:2707–14. 10.1016/j.jcrs.2015.06.038 26796451

[29] Parssinen O Kauppinen M. Associations of reading posture, gaze angle and reading distance with myopia and myopic progression. *Acta Ophthalmol.* (2016) 94:775–9. 10.1111/aos.13148 27369316

[30] Kim JW Eom Y Yoon EG Song JS Jeong JW Park SK Increased near vision spectacle dependence of patients with preoperative myopia after mix-and-match implantation of trifocal EDOF and trifocal IOLs. *J Refract Surg.* (2021) 37:746–53. 10.3928/1081597X-20210802-02 34756137

